# Online adaptive radiotherapy in pelvic and thoracic cancers - comparing toxicities, clinical outcomes and technical parameters between conventional image-guided radiotherapy and online adaptive radiotherapy – the study protocol for the prospective, registry-based cohort study (PRoART)

**DOI:** 10.1186/s12880-026-02622-7

**Published:** 2026-08-05

**Authors:** Laura Anna Fischer, Jann Fischer, Niklas Christian Scheele, Leif Hendrik Droege, Martin Leu, Manuel Guhlich, Jan Tobias Oelmann-Avendano, Andrea Hille, Stephanie Bendrich, Sandra Donath, Olga Knaus, David Alexander Ziegler, Carla Marie Zwerenz, Mahalia Zoe Anczykowski, Hanne Elisabeth Ammon, Pia Franziska Luise Bergau, Charlotta Friederike Pagel, Markus Anton Schirmer, Alina Renata Wenzel, Jasper Frohn, Jona Bensberg, Arne Strauß, Thomas Asendorf, Daniela Schmitt, Stefan Rieken, Rami Atejah El Shafie

**Affiliations:** 1 Department of Radiation Oncology University Medical Center Goettingen (UMG), Robert-Koch-Straße 40, 37075 Goettingen, Germany; 2 Department of Radiation Oncology, University Hospital Hamburg-Eppendorf (UKE), Martinistraße 52, 20246 Hamburg, Germany; 3 Department of Radiation Oncology, Medical University of Lusatia - Carl Thiem, Thiemstraße 111, 03048 Cottbus, Germany; 4 Department of Urology University Medical Center Goettingen (UMG), Robert-Koch-Straße 40, 37075 Goettingen, Germany; 5 Department of Medical Statistics University Medical Center Goettingen (UMG), Humboldtallee 32, 37073 Goettingen, Germany

**Keywords:** Online adaptive radiotherapy, OART, DART, Prostate cancer, Pelvic cancer, Thoracic cancer

## Abstract

**Background:**

Conventional image-guided radiotherapy (IGRT) typically relies on a computed tomography (CT)-based treatment planning process (planning CT, pCT) performed prior to the start of treatment. During this process, a patient-specific treatment plan is generated, which is then delivered to the patient with a linear accelerator on a daily basis, usually with image guidance to compensate for variations in patient setup. However, daily interfractional anatomical variations in target position, shape, and volume, as well as in surrounding organs at risk (OARs), can only be addressed indirectly by adding safety margins, resulting in larger irradiated volumes and potentially increased toxicity. Online adaptive radiotherapy (oART) is a promising and innovative technique to reduce such margins and, consequently, treatment-related toxicity. It uses daily imaging to generate a “plan of the day” that is aligned with the current patient anatomy, often supported by artificial intelligence (AI), while the patient remains on the treatment couch. Through daily image-guided re-optimization of the radiation treatment (RT) plan on the anatomy of the day, target coverage may also be improved. This approach is particularly attractive in the pelvic region, where high interfractional anatomical variability, for example due to peristalsis or changes in bladder and rectal filling, is frequently observed.

**Methods:**

This prospective registry-based cohort study will include patients with pelvic or thoracic tumors with an indication for RT treated with IGRT or oART using the Varian Ethos™ system. The primary endpoint is defined as a 10% reduction in the rate of acute RT related toxicity (≥Common Terminology Criteria for Adverse Events (CTCAE) II°, v5.0) using oART. Secondary endpoints encompass clinical outcomes including late toxicities, tumor control rates, and patient-reported outcomes (PROs), as well as technical factors such as target volume, coverage, dose to OARs and anatomical variability score. While the cohort study compares IGRT versus oART for primary and secondary clinical endpoints, it also evaluates the real oART scenario against two hypothetical control scenarios for technical endpoints.

**Discussion:**

The introduction of oART promises a reduction in toxicities and improved target volume coverage, potentially resulting in enhanced tumor control rates. It is poised to be a pioneering technology in the field of radiation oncology. Given the absence of a direct comparison between IGRT and oART thus far, the prospective registry-based cohort study PRoART aims to address this gap.

**Trial registration:**

Clinical trial number NCT06185062, Clinicaltrials.gov. Registered 12/14/2023. Last update 07/29/2026.

## Background

Approximately 500 000 patients in Germany are diagnosed with malignant neoplasms annually [1]. Radiation oncology is a pillar of oncologic treatment, alongside surgical and pharmacological tumor therapy for many patients. About half of oncology patients receive radiation therapy (RT), typically delivered using medical linear accelerators [2]. A radiation treatment plan is developed based on an initial computed tomography (CT; planning CT, pCT) scan taken before therapy, balancing the dose to the target volume while minimizing exposure to surrounding healthy tissue (organs at risk, OARs). This plan is administered in each treatment session, usually with X-ray-based imaging on the linear accelerator (2D X-ray or 3D Cone-Beam CT, CBCT), followed by positional correction via automated movement of the treatment couch, a process known as Image-Guided Radiotherapy (IGRT). This method only corrects for translational and rotational setup errors by best fitting the static, pre-planned dose distribution of the treatment plan to the daily anatomy, which often requires compromise if organs have shifted in a relevant manner. In contrast, Online Adaptive Radiotherapy (oART) is a procedure in which the radiation plan and corresponding dose distribution are re-optimized on a daily basis to better match the patient’s current anatomy and morphology. Fischer et al. analyzed the impact of oART on prostate bed treatment in a retrospective study of 198 fractions from six patients receiving IMRT, demonstrating a significant improvement in target coverage (D98%) and decreased variability in dose delivery after adaptation [3]. However, to date, only limited published data are available on the clinical benefit of oART, highlighting the need for further prospective studies. A CBCT scan from the linear accelerator before each session provides the basis for this adapted plan, using the initial “old” plan as a reference. This method employs artificial intelligence (AI) to account for both translation and rotation errors, as well as daily interfractional anatomical variations in organ position and size. AI and machine learning are for the first time being used effectively in radiation oncology to reduce treatment planning processes that previously took several days to just minutes. Despite the use of AI, oART requires significant resources in personnel and time compared to IGRT [4]. However, this extra effort is justified by the potential clinical benefits in improved treatment quality. This strategy is particularly promising in the pelvic region due to significant interfractional anatomical variability, such as peristalsis and volume changes in the bladder and rectum.

In recent decades, technological advancements and the implementation of new techniques have significantly reduced the side effects associated with RT. For prostate cancer, Intensity-Modulated Radiotherapy (IMRT) and rotational techniques deliver higher doses to the prostate/prostatic fossa while minimizing exposure to OARs. A prospective, randomized phase III trial “RTOG 0126” showed that IMRT reduced late gastrointestinal (GI) toxicity by 26% compared to 3D-conformal radiotherapy (3D-CRT) [5]. A meta-analysis of 23 studies confirmed that IMRT significantly reduces the incidence of acute GI toxicity by 41% [6]. In other pelvic tumor entities, anatomical variability impacts RT as well. Studies show that oART can significantly improve target volume coverage and reduce OAR dose exposure in primary bladder cancer treatment, potentially lowering acute GI toxicity [7]. Improved sparing of OARs has been shown to reduce therapy-related side effects, as evidenced by studies comparing IMRT with 3D-CRT [8]. In gynecological cancers, oART promises improved target coverage by considering uterine configurations and bladder volumes. Adjuvant radiochemotherapy (RChT) in cervical cancer, delivered using modern IMRT, has shown significantly lower treatment associated toxicity without compromising oncologic outcomes. The randomized phase III trial “PARCER” revealed that, following a median follow-up of 46 months, the cumulative incidence of late grade ≥ II GI toxicity was reduced by half in the IMRT-arm, standing at 21.1% versus 42.2% in 3D-CRT (*p* < 0.001). The cumulative incidence of any late toxicity amounted to 28.1% with IMRT, compared to 48.8% (*p* < 0.001) observed with 3D-CRT [9]. In contrast to pelvic tumors, which often show marked day-to-day anatomical variations due to changing organ volumes, thoracic tumors, such as non-small-cell lung cancer (NSCLC) and small-cell lung cancer (SCLC), exhibit therapy-associated changes like re-opening of atelectasis, resulting in significant anatomical shifts during treatment. Current management involves periodic CT scans and “offline” re-planning [10]. Initial positron-emission tomography (PET)- based planning [11] and additional oART could minimize target volumes and protect healthy tissues. Radiation pneumonitis is a feared side effect, occurring in around 29% of patients after R(Ch)T for NSCLC [12] but recent phase III studies with improved techniques like IMRT have significantly reduced this rate to 0–7.5% [11, 13, 14]. Precision techniques such as IMRT and oART lower toxicity and allow for potential adjuvant immunotherapy in suitable cases [10]. Despite these advancements, reducing acute and chronic side effects remains crucial for patients’ quality of life and treatment decisions.

## Methods and design

### Study design

This clinical study is a prospective and retrospective observational registry-based study that systematically collects data on oART and conventional IGRT in routine clinical practice. Participants are not randomized, nor are they blinded. Three registries will be established due to fundamental differences in diagnosis and treatment among the entities 1) prostate/prostate bed irradiation, 2) pelvic tumors excluding the prostate and 3) thoracic tumors. The primary endpoint is a 10% reduction in RT-associated acute toxicity (Common Terminology Criteria for Adverse Events (CTCAE) v5.0 ≥ Grade II) in the oART cohort compared to the IGRT cohort.

The study comprises two complementary parts: a prospective clinical cohort of patients treated with either oART or IGRT, and virtual sub-cohort in which, for each fraction in the oART arm, the adapted plan is systematically compared with two automatically generated non-adapted reference plans to analyze physical and technical endpoints.

Data collection will be multicentric and will initially take place over six years. Interim analyses are scheduled after 141 patients per cohort, for each registry. The study is endorsed by the working group for Adaptive Radiotherapy of the German Society for Radiation Oncology (DEGRO) and the Radiation Oncology working group (ARO) of the German Cancer Society (DKG). Enrollment of additional centers will be possible during the period of data collection.

### Study objectives

**Fig. 1:**
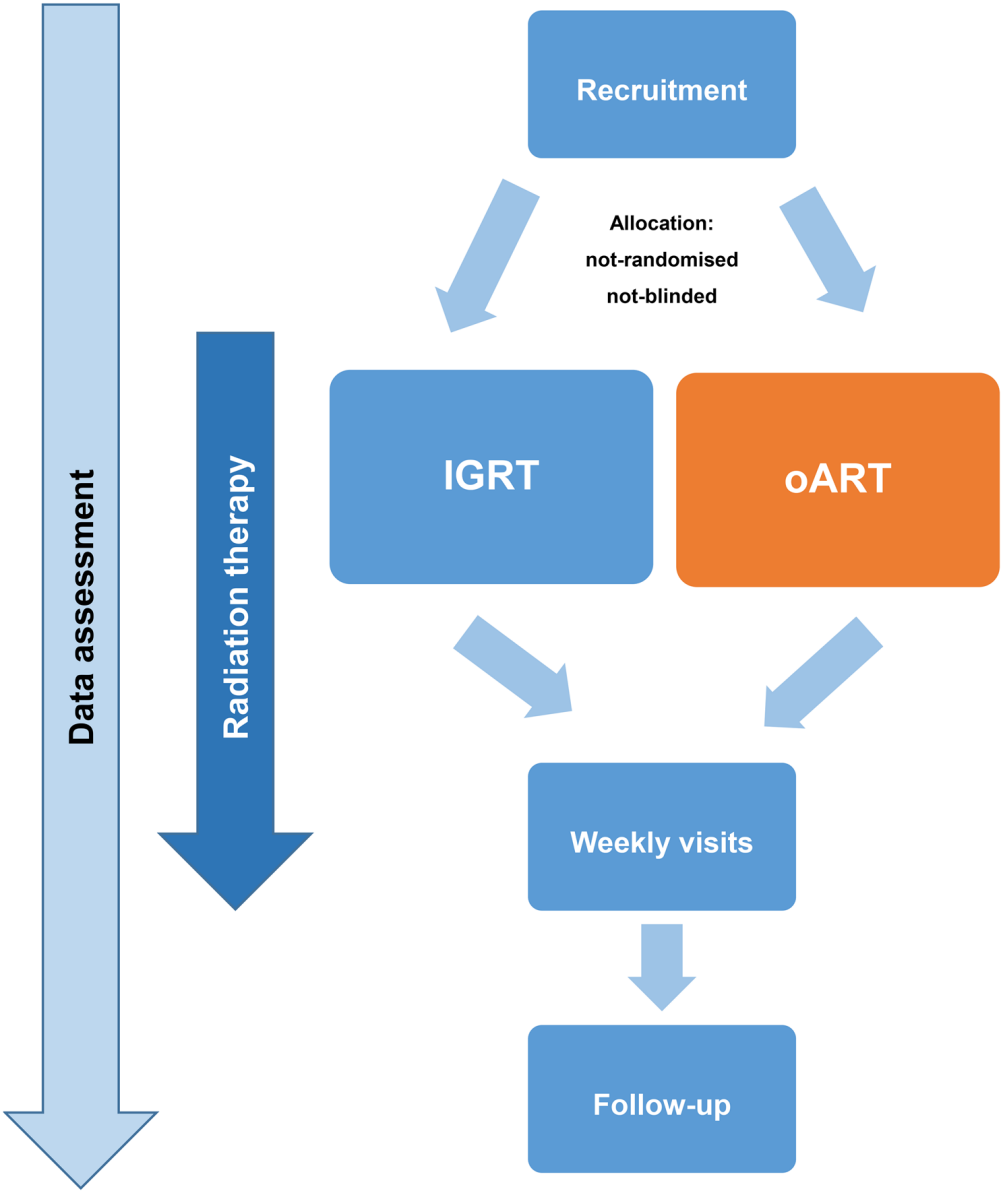
Flow chart of the clinical cohort; legend: IGRT = image guided Radiotherapy; oART = online adaptive radiotherapy

This study centers on the methodology and implementation of oART. Three registries - PRoART-Prostate, PRoART-Pelvis, and PRoART-Thoracic - will be established according to the endpoints outlined in Table 1. Two pragmatic real-world cohorts will be formed in each registry from routine clinical care without randomization nor blinding: the experimental group (oART arm) and the comparison group (IGRT arm) (Fig. 1). Due to capacity limitations at the participating centers, not all patients can receive oART. In routine clinical care, group assignment is primarily determined by the current availability of adaptive therapy time slots and is therefore largely independent of patients’ willingness to participate in the registry-based study. This pragmatic allocation process approximates randomization without formal random assignment.

**Table 1:** Complete list of endpoints and target parameters of PRoART

**Primary endpoint**		**Target parameters**
**Clinical cohort**	10% reduction in acute toxicity using oART	Acute toxicity of CTCAE v5.0 ≥ Grade II and PRO-CTCAE® v1.0 during and within 12 weeks after RT
**Secondary endpoints**		**Target parameters**
**Clinical cohort**	Reduction in late toxicity using oART	Late toxicity of CTCAE v5.0 ≥ Grade II and PRO-CTCAE® v1.0 after RT
	Tumor control	Progression-free survival (PFS) Disease-free survival (DFS) Overall survival (OS) **Only for PRoART-Prostate:** Biochemical failure-free survival (BFFS)
	HRQoL	EORTC QLQ-C30-Score v3.0 **Only for PRoART-Prostate:** EPIC-26 IPSS-Score
	Patient satisfaction	PSQ-18-Score
**Virtual subcohort**	Coverage of target volume	Dose-Volume-Histogram (DVH) of CTV and PTV
	Dose to OARs	DVH of OARs
	Anatomical variability	Dice similarity index
	Treatment time	t_total, IGRT_ resp. t_total, ART_ = Time from first to second CBCT (=time for adaptive process) t_adapt_ = Total time of treatment (=time patient is opened to time patient is closed in software)

An evaluation and testing of the Ethos™ linear accelerator and AI system have already been performed by Varian (Varian Adaptive Intelligence™). The linear accelerator and AI system are certified by the CE (Conformité Européenne) and FDA (Food and Drug Administration, United States).

### Primary endpoint

The primary endpoint is defined as a 10% absolute reduction in the incidence of acute toxicity of grade ≥ II according to CTCAE v5.0, occurring during RT and within twelve weeks after completion of treatment. In addition, we will distinguish between grade II toxicity and toxicity of grade ≥ III. The primary endpoint will be assessed in the clinical cohort (Table 1).

### Secondary endpoints

The secondary endpoints are classified into clinical and technical categories. The clinical cohort will be used to collect secondary clinical endpoints: In addition to systematically recording late toxicities, a comparison of tumor control between the two arms will be performed. Progression-free (PFS), disease-free (DFS), and overall survival (OS) will be assessed. Specifically, for prostate tumors and salvage radiation of the prostate bed, biochemical failure-free survival (BFFS) is considered a surrogate parameter for tumor control and will also be evaluated (Table 1). Treatment response will be assessed according to guideline-based follow-up protocols specific to each tumor entity, using the standard diagnostic procedures recommended in national and international guidelines.

Dose-volume relationships of the target volumes and OARs will be defined as secondary technical endpoints. Additionally, treatment time is a secondary endpoint, defined as the interval between the start of imaging and the beginning of radiation. For secondary technical endpoints, the virtual subcohort (see below) will be used (Table 1).

## Characteristics of participants

### Clinical cohort

All patients with a pelvic or thoracic malignancy and an indication for RT can be included in the data collection. Patients will be assigned to groups (oART arm or IGRT arm) in routine clinical care, primarily based on the current availability of adaptive therapy time slots, thereby approximating random allocation (Fig. 1). Written informed consent is required for all patients prior to study participation. Patients with previous RT to the pelvic or thoracic region are excluded (Table 2). A concurrent cytostatic therapy (RChT) and/or biological therapy is not an exclusion criterion for study participation. Since the treatment is not a study-specific intervention, the decision regarding whether and when to conduct such therapy rests with the multidisciplinary tumor board’s recommendation, which adheres to entity-specific oncological guidelines. Study participation does not influence this decision, the timing of therapy, or the type and dosage of systemic therapy.

**Table 2:** List of eligibility criteria of PRoART

Inclusion criteria	Prostate, pelvic or thoracic cancer Indication for RT Written informed consent Age ≥ 18 years
Exclusion criteria	Previously RT in pelvic or thoracic area (re-irradiation)

### Virtual subcohort

**Fig. 2:**
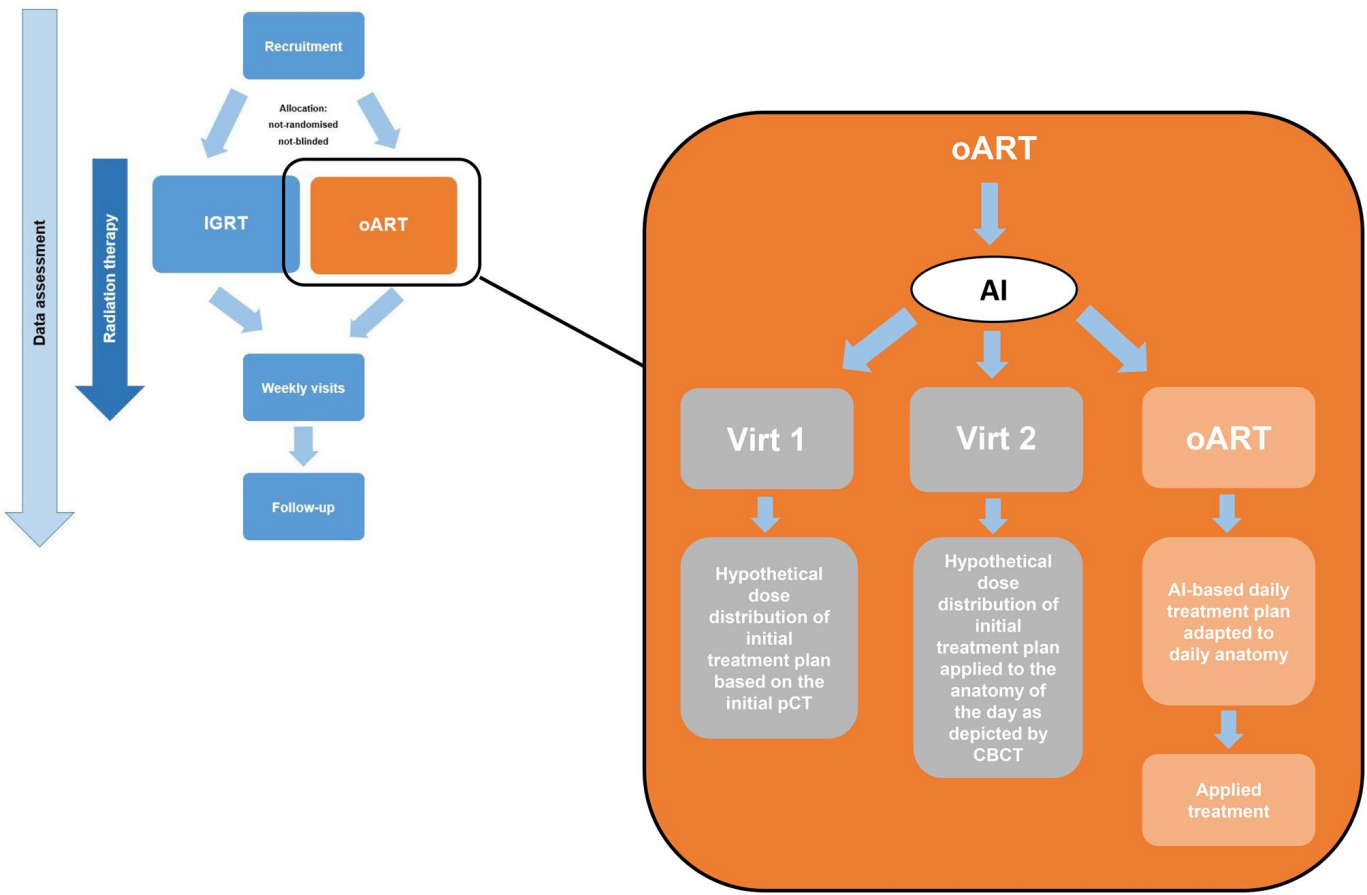
Flow chart of the technical cohort; legend: AI = artificial Intelligence; CBCT = Cone-beam CT; IGRT = image guided Radiotherapy; oART = online adaptive radiotherapy

**Fig. 3:**
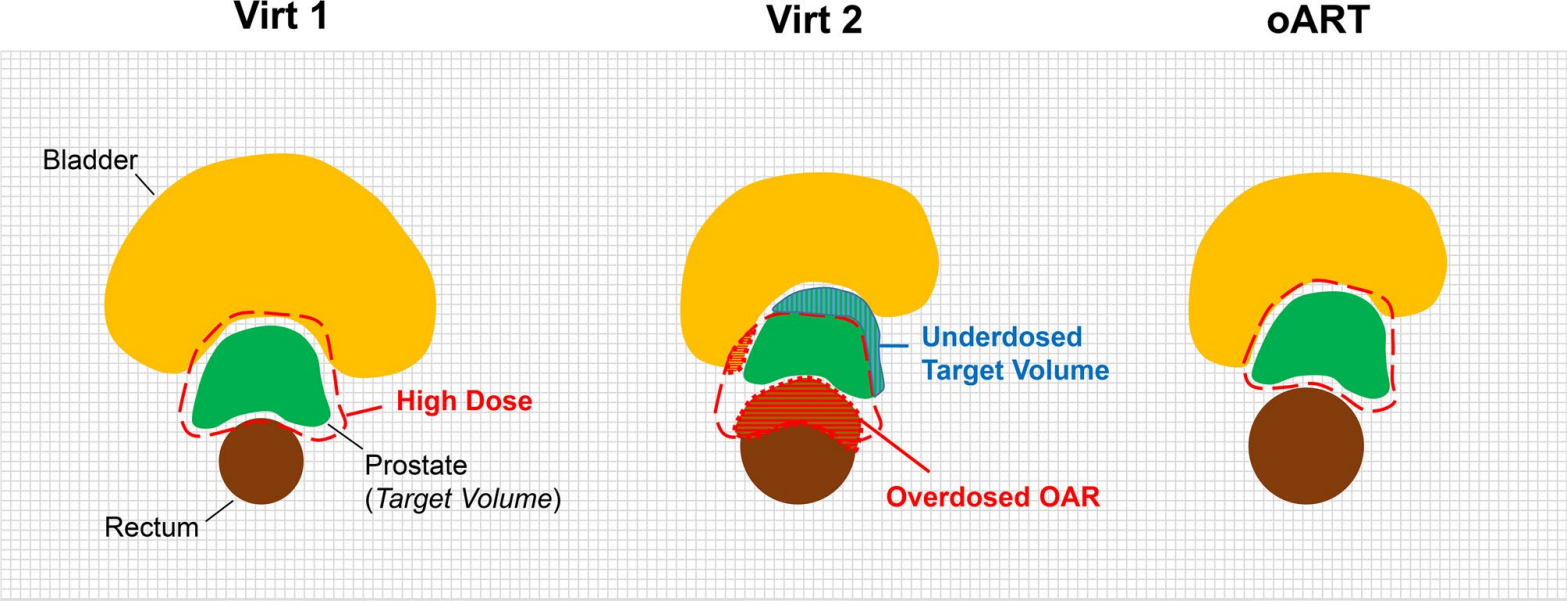
Movement of target volume (e.g. prostate) in the three PRoART-scenarios; legend: virt 1 illustrates the high dose as a marker of the dose distribution in the initial plan on the initial planning computed tomography (CT). Virt 2 displays the initially planned high dose applied to the day’s anatomy as depicted by Cone-beam CT at the medical linear accelerator. This indicates insufficient dose coverage, with underdosing in the target volume (blue) and overdosing in the rectum (red), considered an organ at risk (OAR). Online adaptive radiotherapy (oART) demonstrates how the high dose is adapted to daily anatomical changes, resulting in sufficient coverage without under- or overdosing

Physical and technical endpoints will be assessed in the oART arm. As part of the online adaptation workflow, one actual adapted oART plan and two virtual (hypothetical) non-adapted reference scenarios are automatically generated by the Ethos™ system and presented to the user for evaluation. In our study, these three scenarios are systematically and quantitatively compared to one another; however, for daily treatment, the user can select only between the “Virt 2” and the “oART” plan (Fig. 2):

- Virt 1 – Hypothetical dose distribution of the initial treatment plan (reference plan), based on the initial pCT (Figure 3)
- Virt 2 – Hypothetical dose distribution of the initial treatment plan re-calculated on the anatomy of the day as depicted by CBCT on the treatment linear accelerator (scheduled plan) (Figure 3)
- oART - AI-based daily re-optimization of the treatment plan on the anatomy of the day (adapted plan) (Figure 3)

## Study procedures

The study design follows the typical clinical workflow and execution of a RT course. Patients are included in the PRoART study after being evaluated in the outpatient clinic, informed about the treatment and study process, and providing written informed consent. Initially, baseline data on the tumor entity and its specific characteristics, such as tumor stage, histology, biomarkers, diagnostics, and previous treatment, are collected. Baseline HRQoL is also measured. Study participants are not formally randomized or blinded. The decision to provide the patient with oART on the Ethos™ system is made by the medical team in routine clinical care, primarily based on current capacity for adaptive treatments and patient suitability (for example, tolerance of the relatively long treatment duration and time spent lying on the treatment couch). If no capacity is available at the time of consultation, or if the patient is unsuitable due to their physical condition or comorbidities, the patient is assigned to conventional IGRT.

Both cohorts receive an initial pCT before therapy, on which the corresponding treatment plan is generated, a planning process that may take several days. Both medical and technical personnel are involved, and an individualized treatment plan is generated for each patient through interdisciplinary consultation. This plan is usually a compromise between achieving full target coverage (tumor region ± affected lymph nodes) and minimizing exposure to OARs.

During RT, which is typically administered daily on weekdays over several weeks (depending on the tumor type), a weekly patient visit takes place. Along with assessing acute toxicity, guideline-based supportive therapy can be initiated to alleviate symptoms, if necessary. At the final treatment session, patient satisfaction will be evaluated alongside quality of life in both groups. This assessment aims to determine whether the longer treatment duration and time spent on the treatment couch during oART affect patient satisfaction.

The patients then proceed with guideline-based follow-up care with their specialized oncologists. RT follow-up to monitor long-term toxicity and tumor status is conducted quarterly for the first two years after treatment ends, and then every six months from the third to the fifth year post-treatment.

**Fig. 4:**
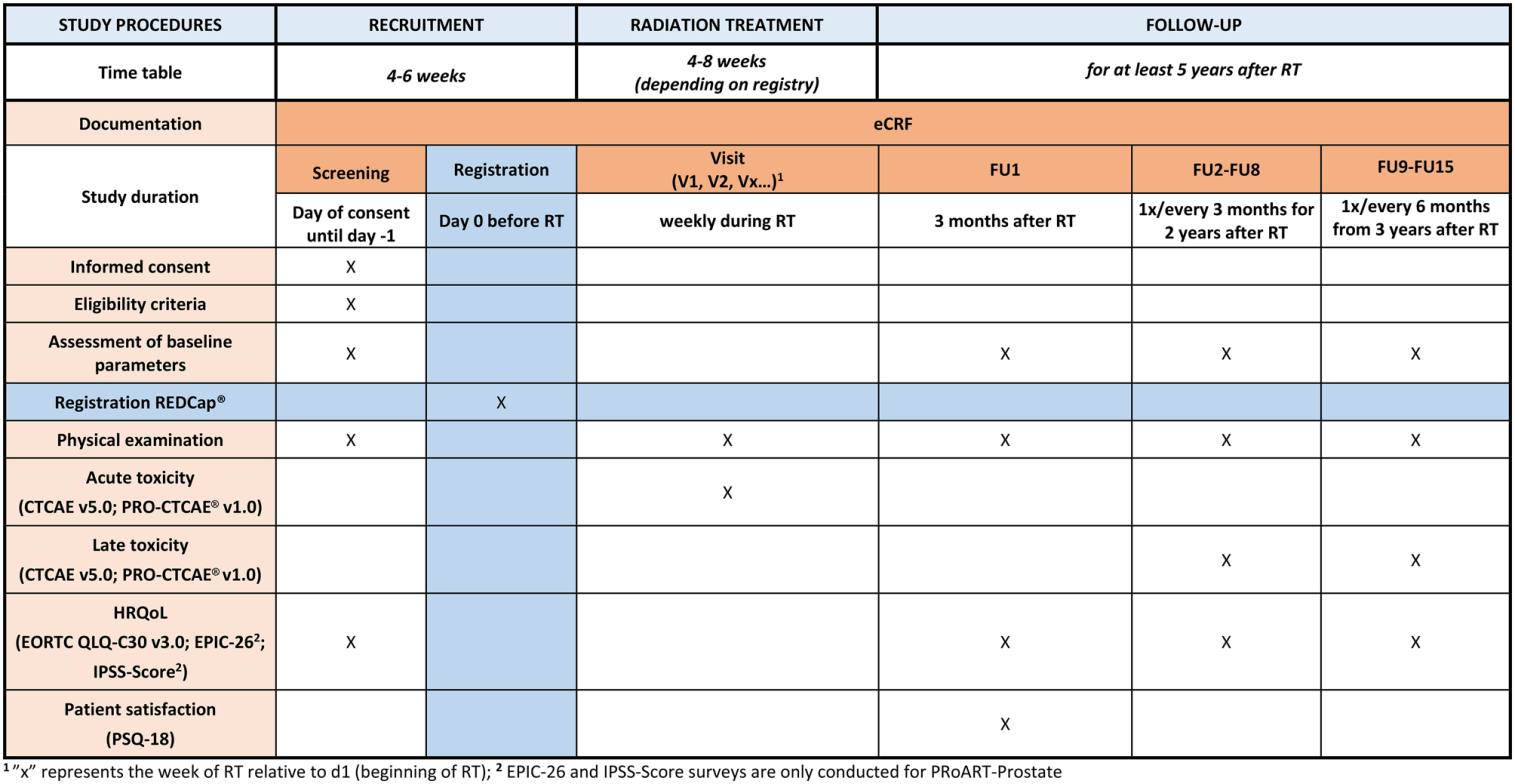
Schedule of assessments of PRoART

Patient-reported baseline data will be collected once before RT, Patient-reported outcomes (PROs) will be collected weekly during therapy and at follow-up visits, and patient satisfaction will be measured once at the end of RT. The standardized questionnaires can be filled out on paper, online via REDCap®, or through a joint phone interview to avoid the need for additional in-person visits. For detailed study procedures and the schedule of assessments, also refer to Fig. 4.

The following validated questionnaires will be used to evaluate clinical endpoints:

- CTCAE v.5.0 to assess acute and late treatment-related toxicity [15]
- PRO-CTCAE® v1.0 to assess HRQoL [16]
- EORTC QLQ-C30 v3.0 to assess HRQoL [17]
- Epic-26 (Expanded Prostate Cancer Index Composite with 26 items) and IPSS (International Prostate Symptom Score) to assess HRQoL in prostate cancer patients [18, 19]
- PSQ-18 (Patient Satisfaction Questionnaire Short Form) to measure patient satisfaction [20]

In addition to the prospectively collected data, the registry will also include data from oART treatments conducted prior to study initiation. These will be collected retrospectively after informing the patients and obtaining their written consent to participate in the study. Retrospective data will be clearly marked and will be analyzed separately to maintain transparency and comparability within the ongoing registry.

## Treatment planning

Radiation treatment planning is performed on a pCT with a maximum slice thickness of 3 mm. The treatment is delivered using a medical linear accelerator and is implemented using IMRT or Volumetric Intensity Modulated Arc Therapy (VMAT). Target volumes and OARs are defined according to the Radiation Therapy Oncology Group (RTOG) contouring guidelines for OARs and internal clinical standard operating procedures (SOPs) for defining clinical target volumes (CTVs), planning target volume (PTVs) margins, and dose concepts. An oART session requires the presence of one radiation oncologist, one medical physicist, and one radiotherapy technologist according to German national guidelines; these requirements may vary in other countries.

## Data management

Data are collected digitally using an electronic case report form (eCRF). All data are pseudonymized and stored in a REDCap® database, a web-based solution meeting all technical Good Clinical Practice (GCP) requirements. Personal data is kept separately in a patient identification protocol containing identification and contact information. The registry database itself contains clinical questionnaire information as well as baseline, therapy, follow-up, and investigation-related outcomes.

## Statistical analysis

The primary endpoint is defined as an absolute reduction in the incidence of acute CTCAE v5.0 grade ≥ II toxicity from 15% to 5% in the oART cohort compared with the IGRT cohort, corresponding to a 10 percentage point absolute reduction. To achieve a power of 80% with a two sided alpha level of 5%, an initial cohort size of *n* = 141 patients per arm (*n* = 282 in total) is required to detect a statistically significant difference (*p* ≤ 0.05) between the oART and IGRT cohorts. The sample size calculation was performed using a chi-squared test for two independent proportions in nQuery (Version 9.5.2). Once *n* = 141 patients per cohort and registry have been reached, an interim analysis of the primary endpoint will be conducted. The sample size calculation was performed in collaboration with the Institute of Medical Statistics at the University Medical Center Goettingen.

For the primary endpoint of acute toxicity and the secondary endpoint of late toxicity, occurrence per patient will be statistically analyzed using general linear models and logistic regression, incorporating baseline covariates like chemo-/immune-/hormone deprivation therapy (yes/no), tumor size, TNM staging/grading/risk scores, and lymph node radiation (yes/no). Proportions will be presented with a 95% confidence interval.

Tumor control and survival rates will be compared between the cohorts using Cox regression, adjusted for covariates, and reported as hazard ratios with 95% confidence intervals.

HRQoL, patient satisfaction, technical endpoints, and other continuous variables will be modeled using mixed linear models to identify marginal group effects, adjusting for relevant baseline covariates such as performance status.

As a registry-based study, patient data will continue to be collected even after the initial analysis of the primary endpoint.

## Study status

The study commenced as a monocentric trial at the University Medical Center Goettingen, and the first patient was enrolled in January 2024. Following approval of the multicenter expansion by the institutional review board on May 22, 2024 (reference 14/9/23), the Department of Radiation Oncology at Jena University Hospital and the Department of Radiation Oncology at Radiologische Allianz GmbH, Hamburg, joined the study. A comprehensive and up-to-date list of participating sites is available at ClinicalTrials.gov (NCT06185062).

## Discussion

Since their implementation, IGRT and IMRT have transformed cancer treatment by improving accuracy and dose distribution, reducing radiation exposure to healthy tissues, and enabling the delivery of higher therapeutic doses to tumors. These techniques have been instrumental in shaping modern radiation oncology, resulting in better patient outcomes [9]. However, challenges have persisted, mainly due to anatomical changes during and between treatment sessions. These variations can lead to missed target volumes and increased toxicity to surrounding healthy tissues. oART seeks to address these issues by re-optimizing treatment plans online based on each patient’s anatomy before every session. By accounting for daily interfractional anatomical variations in patient anatomy and tumor morphology through pre-treatment imaging and online plan re-optimization, oART offers a significant shift towards precision RT and personalized cancer treatment. Some experts predict that its implementation will significantly impact the reduction of safety margins and therapy-related toxicities, surpassing even the advancements brought about by intensity-modulated techniques [8, 21].

Although already in clinical use, the adoption of oART is still limited due to the currently vendor-specific and costly software solutions (for example, Varian Adaptive Intelligence™) as well as due to higher requirements in terms of time and personnel resources. Nevertheless, over the next decade, oART is expected to drive innovation in technical development within RT and radiation oncology. This prospective registry-based study aims to evaluate oART regarding clinical outcomes and technical endpoints, which are closely related and interdependent. The primary endpoint is the reduction of acute toxicity, which is directly related to delivering a lower dose to OARs through daily online plan adaption that accounts for interfractional anatomical variations [7], thereby influencing HRQoL. Therefore, CTCAE v5.0 will be used for an objective assessment, and the PRO-CTCAE® v1.0 catalog will be employed for a subjective, patient-reported perspective. Other clinical endpoints like PFS, DFS and OS potentially reflect improved target volume coverage. Besides oncological outcomes, patient satisfaction and HRQoL also need to be evaluated with regard to the longer time spent on the treatment couch during RT.

A key limitation of this protocol is the lack of formal randomisation, which may lead to selection bias, and the potential imbalance in patient allocation across participating centres. To minimise this risk, patients are assigned based primarily on the availability of adaptive therapy slots and only if they are clinically eligible for both options. Both treatment arms are offered equally, and patients decide after sufficient reflection time. These potential biases will be addressed through detailed baseline data collection, documentation of centre-specific enrolment, and appropriate statistical adjustments, including sensitivity analyses.

In addition, the study also aims to identify prognostic factors that may indicate situations where oART could outperform conventional IGRT. Given the lack of direct comparison between IGRT and oART to date, the prospective registry-based cohort study PRoART aims to address this gap.

## Data Availability

Data generated by this study will be available for access from the corresponding author upon reasonable request.

## Abbreviation

AI: Artificial Intelligence
BFFS: Biochemical failure-free survival
CBCT: Cone-beam CT
CE: Conformité européenne
CT: Computertomography
CTCAE: Common terminology criteria for adverse events v5.0
CTV: Clinical target volume
CRT: Conventional radiotherapy
DFS: Disease-free survival
DVH: Dose-volume histogram
eCRF: Electronic case report form
FDA: Food and drug administration
FU: Follow-up
GCP: Good clinical practice
GI: Gastrointestinal
GU: Genitourinary
HRQoL: Health-related qualitiy of life
IGRT: Image-guided radiotherapy
IMRT: Intensity modulated radiotherapy
NSCLC: Non-small-cell lung cancer
oART: Online adaptive radiotherapy
OARs: Organs at risk
OS: Overall survival
pCT: Planning CT
PET: Positron-emission tomography
PFS: Progression-free survival
PTV: Planning target volume
PRO: Patient-reported outcomes
PRoART: Pelvic cancer registry for online adapted radiotherapy
PRO-CTCAE®: Patient-Reported outcomes of the common terminology criteria for adverse events v1.0
RChT: Radiochemotherapy
RT: Radiation therapy, radiotherapy, radiation treatment
RTOG: Radiation therapy oncology group
SCLC: Small-cell lung cancer
SOP: Standard operating procedure
VMAT: Volumetric intensitiy modulated arc therapy
